# A2 Noradrenergic Lesions Prevent Renal Sympathoinhibition Induced by Hypernatremia in Rats

**DOI:** 10.1371/journal.pone.0037587

**Published:** 2012-05-21

**Authors:** Gustavo Rodrigues Pedrino, André Henrique Freiria-Oliveira, Débora Simões Almeida Colombari, Daniel Alves Rosa, Sergio Luiz Cravo

**Affiliations:** 1 Department of Physiological Science, Universidade Federal de Goiás, Goiânia, Goiás, Brazil; 2 Department of Physiology and Pathology, School of Dentistry, São Paulo State University, Araraquara, São Paulo, Brazil; 3 Department of Physiology, Escola Paulista de Medicina, Universidade Federal de São Paulo, São Paulo, São Paulo, Brazil; The University of Manchester, United Kingdom

## Abstract

Renal vasodilation and sympathoinhibition are recognized responses induced by hypernatremia, but the central neural pathways underlying such responses are not yet entirely understood. Several findings suggest that A2 noradrenergic neurons, which are found in the nucleus of the solitary tract (NTS), play a role in the pathways that contribute to body fluid homeostasis and cardiovascular regulation. The purpose of this study was to determine the effects of selective lesions of A2 neurons on the renal vasodilation and sympathoinhibition induced by hypertonic saline (HS) infusion. Male Wistar rats (280–350 g) received an injection into the NTS of anti-dopamine-beta-hydroxylase-saporin (A2 lesion; 6.3 ng in 60 nl; n = 6) or free saporin (sham; 1.3 ng in 60 nl; n = 7). Two weeks later, the rats were anesthetized (urethane 1.2 g⋅kg^−1^ b.wt., i.v.) and the blood pressure, renal blood flow (RBF), renal vascular conductance (RVC) and renal sympathetic nerve activity (RSNA) were recorded. In sham rats, the HS infusion (3 M NaCl, 1.8 ml⋅kg^−1^ b.wt., i.v.) induced transient hypertension (peak at 10 min after HS; 9±2.7 mmHg) and increases in the RBF and RVC (141±7.9% and 140±7.9% of baseline at 60 min after HS, respectively). HS infusion also decreased the RSNA (−45±5.0% at 10 min after HS) throughout the experimental period. In the A2-lesioned rats, the HS infusion induced transient hypertension (6±1.4 mmHg at 10 min after HS), as well as increased RBF and RVC (133±5.2% and 134±6.9% of baseline at 60 min after HS, respectively). However, in these rats, the HS failed to reduce the RSNA (115±3.1% at 10 min after HS). The extent of the catecholaminergic lesions was confirmed by immunocytochemistry. These results suggest that A2 noradrenergic neurons are components of the neural pathways regulating the composition of the extracellular fluid compartment and are selectively involved in hypernatremia-induced sympathoinhibition.

## Introduction

Several lines of evidence have found that acute or chronic hypernatremia engages several effector natriuretic mechanisms that remain active until the reestablishment of the plasma sodium concentration. [1]–[8]. Accordingly, previous studies have demonstrated that the intravenous infusion of hypertonic saline (HS) increases arterial blood pressure and renal vascular conductance and induces regionally distinct changes in the sympathetic nerve activity, such as increases in lumbar and decreases in renal and splanchnic nerve discharge [2]–[4], [8], [9]. It is generally accepted that the inhibition of renal sympathetic nerve activity (RSNA) is a key component of these adjustments as it can produce renal vasodilation, decrease renin secretion and reduce renal sodium reabsorption, all mechanisms that, either in isolation or synergistically, will lead to massive sodium loss. However, the central aspects of the neural pathways involved in the activation of these systems remain unclear.

Recent studies have demonstrated that A2 noradrenergic neurons located within intermediate and caudal levels of the nucleus of the solitary tract (NTS) are involved in body fluid homeostasis and cardiovascular regulation [10]–[14]. Changes in the volume and composition of the extracellular compartment stimulate the expression of c-Fos and FosB in A2 neurons, indicating their activation [12], [15]. In addition, Duale *et al.* [10] demonstrated that the silencing of A2 neurons affected water consumption and urine output.

Neuroanatomical studies have shown that A2 noradrenergic neurons receive sensory afferent input from the cardiovascular and gastrointestinal systems [16], [17]. These noradrenergic neurons project throughout the central nervous system, including areas related to the cardiovascular system and to the regulation of the hydroelectrolytic balance, such as the median preoptic nucleus (MnPO), the subfornical organ (SFO), the paraventricular nucleus (PVN), and the supraoptic nucleus (SON) [18]–[21]. Overall, this evidence suggests an important role of A2 neurons in the maintenance of the circulating volume.

Therefore, in the present study, we tested the hypothesis that the renal sympathoinhibition and vasodilation, induced by acute increases in the plasma sodium levels, depend upon the integrity of A2 neurons. We used anti-dopamine β-hydroxylase (DβH) saporin to induce lesions in the noradrenergic neurons in the NTS and measured the effects of these lesions on the blood pressure pressor, renal vascular conductance, and sympathetic responses induced by acute hypernatremia.

## Results

### Lesion of the A2 noradrenergic neurons with nanoinjections of anti-DβH–saporin into the NTS

**Figure 1:**
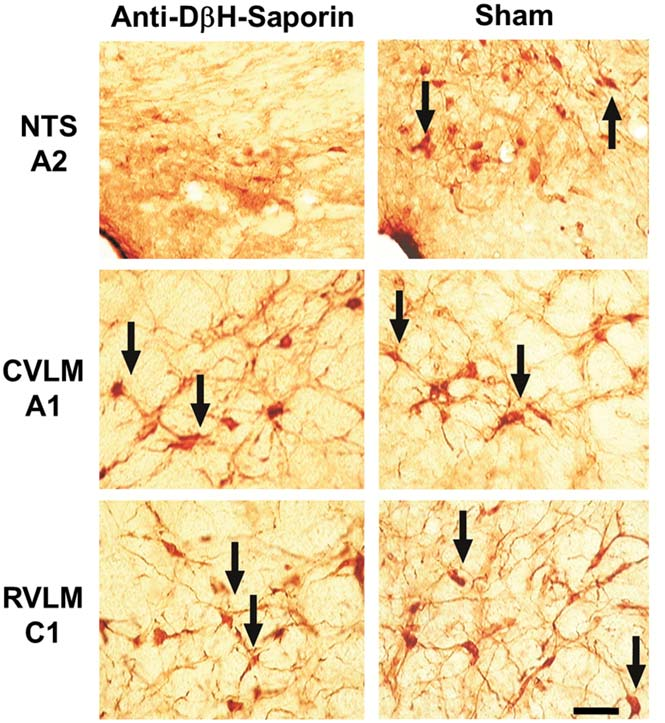
Medullary catecholaminergic neurons. Photomicrographs taken at 3 levels of the medulla showing TH-immunoreactive cells in the NTS (A2 noradrenergic neurons), CVLM (A1 noradrenergic neurons) and RVLM (C1 adrenergic neurons) in rats nanoinjected with unconjugated saporin (sham) or anti-DβH-saporin. Arrows indicate TH-positive cells. Scale bar 50 µm.

**Figure 2:**
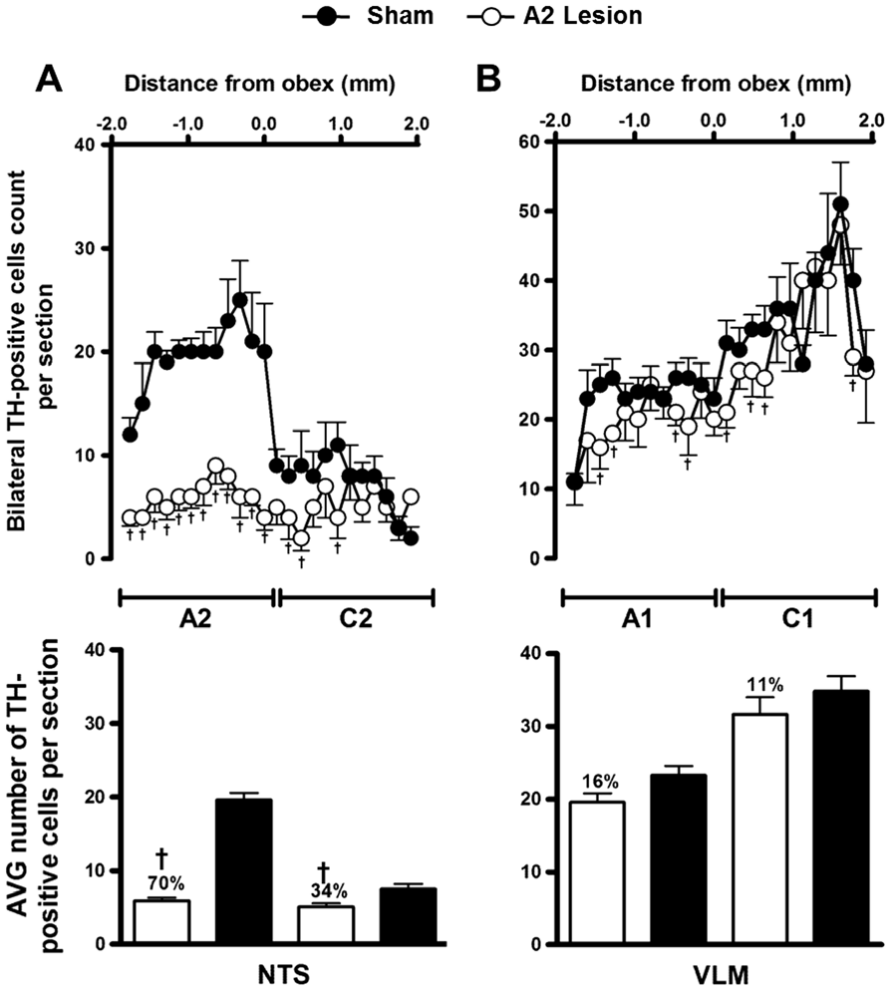
Lesion of A2 noradrenergic neurons with nanoinjections of anti-DβH-saporin into the NTS. Number and average (mean ± S.E.M.) of TH-positive cells in 40-µm-thick sections from the dorsal (A) and the ventrolateral medulla (B). Sections were taken from 1.9 mm rostral to the obex to 1.9 mm caudal to the obex in animals submitted to A2 or sham lesions. Bilateral nanoinjections of anti-DβH-saporin into the NTS produced a loss of TH-containing neurons in this area (A2 group, loss = 70%; C2 group, loss = 34%), in the RVLM (C1 group, loss = 11%) and in the CVLM (A1 group, loss = 16%). † p<0.05 compared with sham lesion.

Tyrosine hydroxylase (TH)-positive neurons were found within the ventrolateral medulla (VLM) and the NTS from positions approximately 1800 µm caudal to 1900 µm rostral to the obex (Figures 1 and 2). In the saporin-treated animals (sham; n = 7), TH-positive neurons in the region between the obex and 1800 µm caudal to the obex averaged approximately 20 cells per section (A2 neurons). In rats treated with bilateral nanoinjections of anti-DβH-saporin into the NTS (n = 6), the number of TH-positive neurons caudal to the obex was reduced to approximately six cells per section. Overall in this region, which encompasses the area of the A2 noradrenergic neurons, the number of TH-positive neurons was reduced by 70% (range 66 to 88%) compared to the saporin-treated animals (Figures 1 and 2).

The nanoinjection of anti-DβH-saporin into the NTS reduced the number of TH-positive neurons in the C2 adrenergic cell group. Within the region located from the obex up to 1900 µm rostral (the region encompassing the C2 cells), a reduction of approximately 34% in the number of TH immunopositive neurons was observed (Figures 1 and 2). Slight reductions in the total number of TH-positive neurons were also observed in the A1 and C1 cell groups in the VLM, although they did not reach statistical significance. In the region from 1800 to 300 µm caudal to the obex, A1 noradrenergic neurons were reduced by 16% (p = 0.0591; Figures 1 and 2). Similarly, in the region from 300 µm caudal to 1900 µm rostral to the obex, C1 adrenergic neurons were reduced by 11% (p = 0.3296; Figures 1 and 2).

### Effects of HS infusion on plasma sodium and blood hemoglobin concentrations

The plasma sodium and hemoglobin concentration changes that were induced by HS infusion were determined in sham and A2-lesioned rats. The pre-infusion plasma sodium concentrations were similar in sham (141±1.2 mM; n = 4) and A2-lesioned rats (141±2.2 mM; n = 4). The plasma sodium levels increased similarly after HS infusion in both groups (150±1.4 mM and 149±1.2 mM in sham and A2-lesioned rats, respectively, 10 min after infusion) and remained at these levels throughout the experimental period (148±1.2 mM and 150±1.7 mM 60 min after HS infusion). Ten minutes after HS infusion, there were no changes in the blood hemoglobin concentrations in sham (−1±1.0%) and A2-lesioned rats (−1±0.9%). The blood hemoglobin concentration remained at these levels throughout the experimental period (−1±0.6% and −1±0.8%, respectively, 60 min after HS infusion).

### Effects of A2 noradrenergic neuron lesions on cardiovascular and RSNA responses induced by HS infusion

The body weight and baseline MAP, HR, RBF, RVC and RSNA are shown in Table 1. These variables were similar in sham rats and A2-lesioned rats.

**Table 1:** Baseline values for body weight, MAP, HR, RBF, RVC and RSNA in sham and A2 lesioned rats.

Group	b.w.g	MAPmm Hg	HRbpm	RBFml⋅min^−1^	RVCµl(min⋅mmHg)^−1^	ƒRSNAmV
**Sham**	319±6	123±4	346±15	3.3±0.2	26.7±1.1	185±14.6
**A2 lesion**	323±5	123±2	326±15	3.1±0.5	25.0±4.1	183±28.1

Values are means ± S.E.M. b.wt., body weight; MAP, mean arterial pressure; HR, heart rate; RBF, renal blood flow; RVC, renal vascular conductance; ∫RSNA, integrate of renal sympathetic nerve activity.

**Figure 3:**
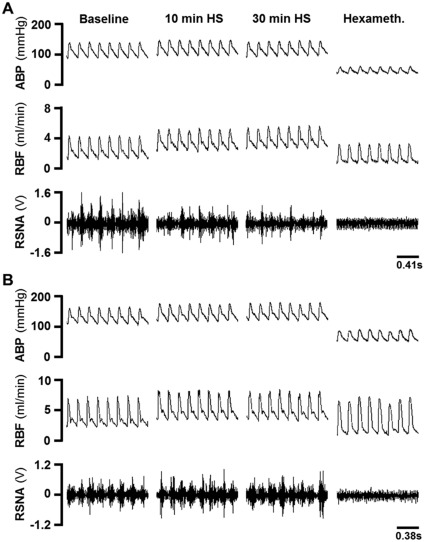
Typical examples. Digitized record of cardiovascular and sympathetic responses induced by hypertonic saline infusion in sham (A) and A2-lesioned rats (B). ABP = arterial pressure, RBF = renal blood flow, RSNA = renal sympathetic nerve activity (RSNA), hexameth. = hexamethonium.

**Figure 4:**
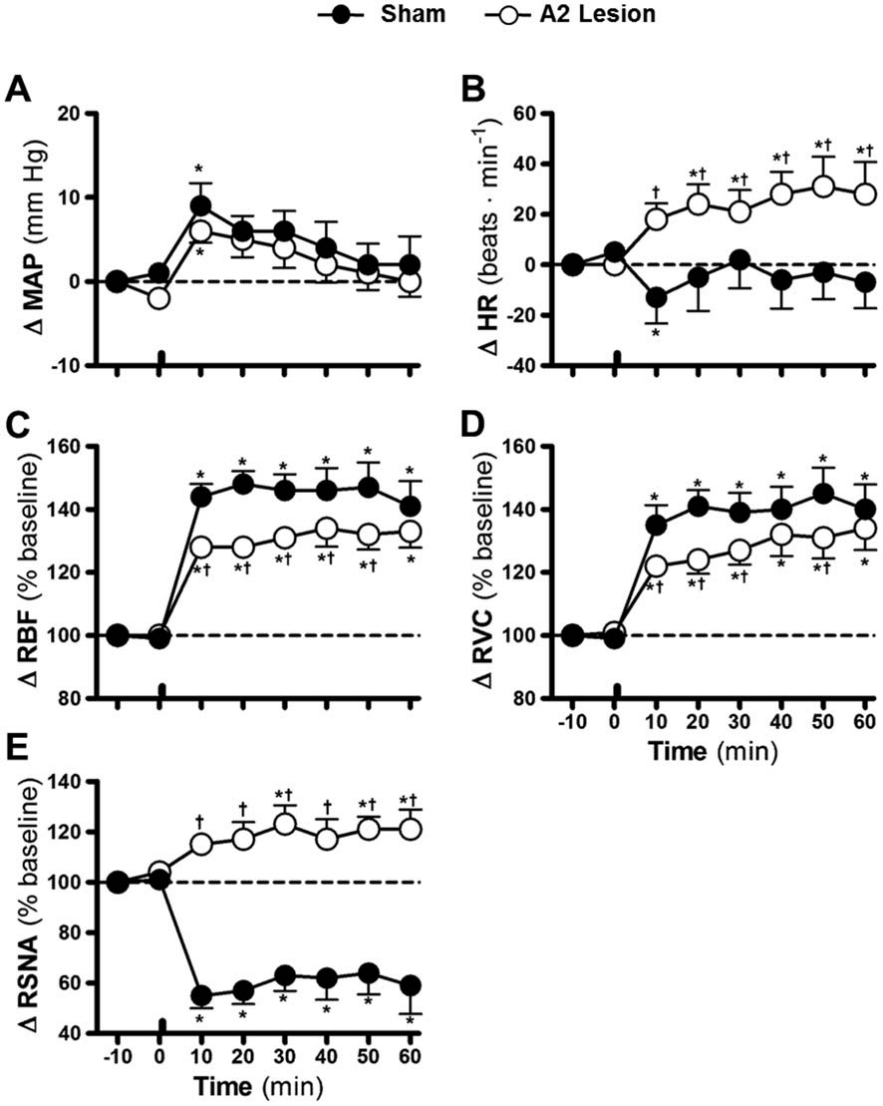
Effects of A2 noradrenergic neuron lesions on cardiovascular and autonomic responses induced by hypernatremia. Effects of infusion of hypertonic saline (3 M NaCl, 1.8 ml·Kg^−1^ body weight) on mean arterial pressure (Δ MAP; A), heart rate (Δ HR; B), renal blood flow (Δ RBF; C), renal vascular conductance (Δ RVC; D) and renal sympathetic nerve activity (Δ RSNA; E) in sham and A2-lesioned rats. The bars indicate the moment of hypertonic saline infusion. Error bars indicate S.E.M. * p<0.05 compared with baseline; † p<0.05 compared with sham lesion.

In sham animals, the infusion of HS increased MAP by 9±2.7 mmHg 10 min after infusion (Figures 3A and 4A). MAP returned to basal levels 20 min after the HS infusion (6±1.8 mmHg of baseline values; Figures 3A and 4A). In sham animals, a discrete reduction in the heart rate 10 min after the HS infusion (−13±10.3 beats•min^−1^ of baseline values; Figures 3A and 4B) was observed. Ten minutes after the HS infusion, the RBF and RVC increased to 144±4.0 and 135±6.3% of baseline values, respectively, and they remained augmented after 60 min (141±7.9% and 140±7.9% of baseline values, respectively; Figures 3A, 4C–D). The reduction in the RSNA reached a nadir approximately 10 min after the HS infusion (−45±5.0% of baseline values; Figures 3A and 4E) and remained lower for the remainder of the experimental period (−41±11.3% of baseline values at 60 min after HS infusion; Figures 3A and 4E).

In A2-lesioned rats, the pressor response induced by the HS infusion was similar to that of the sham group (6±1.4 mmHg above baseline at 10 min after HS infusion; Figures 3B and 4A). A sustained increase in the HR was also observed 10 min after the HS infusion (18±6.3 beats min^−1^ of baseline values; Figures 3B and 4B). The RBF and RVC increased after the HS infusion (128±2.5% and 122±2.2% of baseline values 10 min after HS infusion; Figures 3B and 4C–D) and remained augmented after 60 min (133±5.2% and 134±6.9% of baseline values; Figures 3B and 4C–D). However, these increases were slightly lower than those observed in the sham animals. In A2-lesioned rats, the reduction in the RSNA was abolished and reversed to a slight increase after the HS infusion (15±3.1% of baseline levels 10 min after HS infusion; Figures 3B and 4E); this difference reached statistical significance beginning at 30 min after the HS infusion.

### Effects of A2 noradrenergic neuron lesions on baroreceptor reflexes

**Figure 5:**
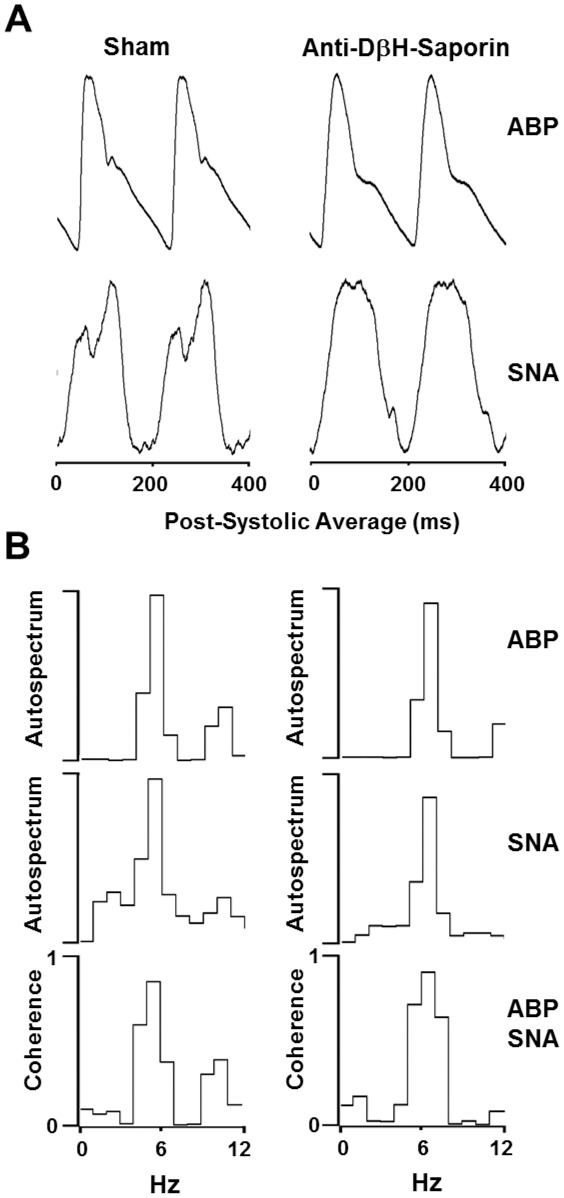
Effects of A2 noradrenergic neuron lesions on baroreceptor reflexes. Post-systolic averages of arterial pressure (AP) and renal sympathetic nerve activity (SNA) waves (A) and coherence values between AP and SNA power spectrum (B) before hypertonic saline infusion in sham and A2-lesioned rats.

The functionality of the baroreceptor reflexes was evaluated by the analysis of the cardiac-related components of the RSNA and the coherence between the AP and RSNA. In A2-lesioned rats, RSNA bursts were synchronized with the cardiac cycle, as demonstrated by PAP-triggered averaging of the RSNA (Figure 5A). The RSNA power spectra displayed a peak that corresponded to the HR, indicating a prominent role of the baroreceptor reflexes in modulating spontaneous RSNA discharge. In these animals, a correlation between the RSNA bursts and the cardiac cycle led to high AP-RSNA coherence values (mean 0.83±0.04; Figure 5B). Similar results were observed in the A2-lesioned rats, as shown by the synchronization of the RSNA to the cardiac cycle (Figure 5A), a prominent peak in the power spectra corresponding to the HR, and high AP-SNA coherence (0.85±0.04; Figure 5B).

## Discussion

Previous studies have indicated that A2 neurons are involved in the regulation of body fluid homeostasis [10]–[14], [22]–[25]. However, there was previously no evidence to support the contribution of these neurons to the autonomic control of the circulating volume composition. The present study provides a key new observation: lesions of the A2 noradrenergic cluster reduced HS-induced renal vasodilation. Moreover, in A2-lesioned rats, hypernatremia failed to decrease renal sympathetic activity during the observation period. Together, these results point out the involvement of A2 noradrenergic neurons in the autonomic responses that are induced by acute changes in the plasma sodium concentration, and the results specifically highlight their involvement in the regulation of renal sympathetic nerve activity.

Several studies have aimed to quantitate and validate the specificity of neuronal lesions induced by the central nanoinjection of anti-DβH-saporin, and the results obtained are compelling. Madden et al. [26] demonstrated that the nanoinjection of anti-DβH-saporin into the C1 adrenergic neuron rostral cluster, located in the rostroventrolateral medulla (RVLM) region, promoted lesion of these neurons as well as lesions of A1 and A5 catecholaminergic neurons. In addition to demonstrating that the lesion effect is caused by the uptake of synaptic terminals, these authors demonstrate that the anti-DβH-saporin could damage noradrenergic and adrenergic neurons located in the nanoinjection site itself. It is postulated that, in these neurons, the somatodendritic exocytosis of catecholamines resulted in the exposure of DβH and therefore the possible uptake of the anti-DβH-saporin complex via their soma and/or dendrites. In agreement with these results, we have recently demonstrated that the nanoinjection of anti-DβH-saporin into the caudoventrolateral medulla (CVLM) region promoted specific lesions to the A1 cluster located around the center of the nanoinjections [3], [27]. As noted earlier, the current study also shows that the nanoinjection of anti-DβH-saporin in the NTS promoted, on average, a reduction of approximately 70% in all of the catecholaminergic cells compared to the control cluster (nanoinjection of saporin) of A2 noradrenergic neurons.

An important aspect to be considered is whether nanoinjections of anti-DβH-saporin into the A2 area destroy the DβH-containing cells outside of the NTS and if the results observed may be attributed to these remote lesions. Our results indicate that nanoinjections of anti-DβH-saporin into the NTS/A2 area induced a discrete (16%) reduction in the total number of TH-positive cells in the CVLM/A1 cluster and an 11% reduction in the RVLM/C1 cluster. It is likely that these lesions result from the uptake of the conjugated toxin by efferent projections from the ventrolateral medulla to the A2 region of the NTS. Studies performed in our laboratory have demonstrated that lesions of less than 32% of the A1 noradrenergic cells produced by nanoinjections of anti-DβH-Saporin into the CVLM were ineffective in altering the renal sympathoinhibition induced by sodium overload (G.R.P. & S.L.C, unpublished observations). Consistent with these results, Madden & Sved [28] demonstrated that sympathoexcitatory responses induced by baroreceptor unloading or chemoreceptor activation were abolished only in animals with lesions in more than 80% of the C1 catecholaminergic group. These results suggest that extensive lesions of cell groups are necessary to induce significant and reproducible effects. Therefore, it is unlikely that the lack of renal sympathoinhibition in the A2-lesioned rats in our study may be due to the less than 20% lesions that were found in the A1/C1 areas.

Previous studies have attempted to establish the effects of hypernatremia on cardiovascular and renal parameters. Similar to the results obtained in the present study, they describe sustained increases of blood flow and renal vasculature conductance, combined with transient hypertension and bradycardia [2], [3], [8], [9], [27], [29], [30]. It is assumed that these adjustments are triggered by an increase in the sodium plasma concentration, part of an integrated set of responses to promote natriuresis and thus restore the regular volume conditions. Moreover, we have previously demonstrated that the vasodilation induced by sodium overload is specific to the renal bed, as there is no change in the aortic or mesenteric vascular conductance [9]. Evidence suggests the renal vasodilation induced by sodium overload seems to involve neural and humoral components, including a reduction of the renal sympathetic nerve activity and the release of vasoactive substances, such as ANP [2], [3], [5], [8], [31], [32].

Studies have shown the importance of medullary noradrenergic neurons in the integration of the mechanisms involved in the autonomic, cardiovascular and respiratory systems [3], [10]–[15], [22], [24], [25], [27], [33]–[36]. With regard to the A1 noradrenergic cluster, several authors have shown that these neurons are important for the response induced by changes in the circulating volume [3], [12], [15], [22], [27], [33]–[37]. Studies have demonstrated that increases in the sodium plasma concentration induce the expression of c-Fos in these noradrenergic neurons, indicating their activation [15], [23]. Recently, we have shown that specific lesion of the A1 noradrenergic cluster abolishes the vasodilation and the renal sympathoinhibition that are induced by hypernatremia [3], [27]. These results indicate that A1 neurons are involved in both neural and humoral mechanisms in response to hypernatremia, once their specific lesion abolishes the renal sympathoinhibition and vasodilation that are induced by the sodium overload.

With regard to the A2 cluster of noradrenergic neurons, recent evidence has shown that the viral inactivation of these neurons decreased the daily water intake [10], showing their involvement in the behavioral regulation of the circulating volume. Other studies on the expression of immediate early genes show that these noradrenergic neurons located in the NTS are activated by increases in the osmolality or plasma volume [12], [23], [24]. Extracellular neural activity recordings have identified NTS neurons in which activity increases after sodium overload [24]. The present study shows that the specific lesion of A2 neurons abolishes the renal sympathoinhibition induced by the infusion of hypertonic saline. To the best of our knowledge, no other study has yet demonstrated the participation of these neurons in the autonomic component of the response to hypernatremia. In addition, we have also demonstrated that the renal vasodilation induced by sodium overload was not abolished but was reduced in animals with A2 lesions, suggesting that the humoral component of the response to hypernatremia is intact in these animals. Therefore, while A1 noradrenergic neurons appear to modulate both the endocrine and neural components that are observed in response to hypernatremia, the A2 noradrenergic neurons seem to be specifically related to the modulation of renal sympathetic activity.

Anatomical studies have shown that medullary noradrenergic neurons receive sensory information from baro- and cardiopulmonary receptors [16], [24], [34], [38], [39]. From medullary catecholaminergic and non-catecholaminergic neurons, the information about the circulating volume would be processed and transmitted to hypothalamic centers involved in the regulation of osmolality. In fact, both A1 and A2 catecholaminergic groups project densely to hypothalamic targets as the PVN and MnPO [18], [20], [21], [40]–[43]. The main difference is that, whereas A1 neurons project densely to both magno- and parvocellular regions of the PVN, A2 neurons project primarily to the dorsal and ventral parvocellular groups of this nucleus [41]. These anatomical data are compatible with the idea that A2 neurons may be selectively involved with the neural regulation of sympathetic activity in response to acute hypernatremia, whereas A1 neurons may be involved in the regulation of both neuronal and humoral components.

The noradrenergic neurotransmission in the hypothalamic regions appears to play a significant role in the responses induced by changes in the circulating volume. Data have shown that the nanoinjection of α-adrenergic-antagonists into the AV3V region reduces the hypertension response and secretion of ANP and oxytocin induced by sodium overload [44], [45]. Similarly, recent studies conducted by our laboratory have shown that the blockade of α_1_ or α_2_ adrenoceptors located in the MnPO abolishes the renal vasodilation induced by hypertonic saline infusion [4]. Thus, the lesion of medullary noradrenergic neurons and the α adrenoceptor blockade in the MnPO nucleus change the cardiovascular outcome in response to hypernatremia, supporting the idea that ascending projections from medullary noradrenergic neurons and the MnPO nucleus mediate the endocrine and autonomic adjustments involved in this response.

Together, the results obtained in this study are consistent with the hypothesis that the A2 noradrenergic neurons activated by peripheral afferents are part of the central pathways modulating the adjustments induced by acute changes in osmolality. Thus, as these noradrenergic neurons are part of the pathways involved in responses to changes in the plasma sodium concentration, a dysfunction in these cells would result in the inefficient operation of these adjustments, which could contribute to the development of diseases such as arterial hypertension.

## Materials and Methods

### Animals

All experiments were conducted on adult male Wistar rats (280–350 g). The animals came from the central animal house at the Universidade Federal de São Paulo. All protocols described here were approved by the Medical Ethics Committee at the Universidade Federal de São Paulo (protocol # 0739/06) and were performed in strict accordance with the Guidelines for Care and Use of Laboratory Animals of the National Health Institute.

### Nanoinjections of anti-DβH-saporin or saporin into the NTS

The animals were anaesthetized with halothane (2–3% in 100% O_2_) and mounted prone on a stereotaxic apparatus (David Kopf Instruments, Tujunga, CA, USA) with the incisor bar 11 mm below the interaural line. After partial removal of the occipital bone, the meninges covering the dorsal surface of the brainstem were cut and retracted, and the calamus scriptorius was visualized. Nanoinjections of anti-DβH-saporin (6.3 ng in 60 nl; Advanced Targeting Systems, San Diego, CA, USA) or an equimolar dose of saporin (1.3 ng in 60 nl; Advanced Targeting Systems, San Diego, CA, USA) were made bilaterally at two levels of the NTS. For all nanoinjections into the NTS, a glass micropipette was positioned as follows: −0.5 and −0.0 mm caudal to the *calamusscriptorius*, 0.0 mm lateral to the midline, and 0.3 mm ventral to the dorsal surface. These coordinates were based on the region of the NTS comprising the A2 noradrenergic group [46]. After nanoinjection, the micropipette was left in place for 3–5 min. The incision was then closed, and the animals were placed on a heated pad to maintain their body temperature during recovery. Penicillin (60 000 IU kg^−1^ b.wt., i.m.; Sigma-Aldrich, St. Louis, MO, USA) was injected for prophylaxis after the surgery. The animals were studied for 15–25 days after the nanoinjections into the NTS.

### Surgical procedures

On the day of experiments, the rats were anesthetized with halothane (2–3% halothane in 100% O_2_), and the catheters were inserted into the right femoral vein and artery. The right jugular was also dissected, and a catheter was placed closer to the right atrium for the HS infusion (see below). After the catheter placement, the rats were removed from the halothane, and the anesthesia was maintained with urethane (1.2 g⋅kg^−1^ b.wt., i.v.; Sigma-Aldrich, St. Louis, MO, USA). The trachea was cannulated to reduce airway resistance, and the rats were mounted prone on a stereotaxic apparatus (David Kopf Instruments) with the bite bar set at 3.4 mm below the interaural line. The postganglionic renal sympathetic nerve activity (RSNA) was recorded from the left renal nerve with bipolar platinum electrodes in a monopolar configuration. In experiments measuring the renal blood flow (RBF), miniature ultrasonic transit-time flow probes (Transonic Systems Inc., Ithaca, NY, USA) were placed around the left renal artery. The body temperature was kept at 37±0.5°C with a thermostatically controlled heated table.

### Recording of arterial pressure, heart rate, and renal blood flow

To register the blood pressure, the arterial catheter was connected to a pressure transducer attached to a bridge amplifier. The pulsatile pressure was recorded continuously with an MP150 analog-to-digital converter (Biopac Systems, Inc., Goleta, CA, USA). The mean arterial pressure (MAP) and heart rate (HR) were determined through the pulsatile signal with AcqKnowledge software (version 3.7.1.; Biopac Systems, Inc., Goleta, CA, USA). To measure the RBF, a flow probe was connected to an ultrasonic transit-time flowmeter (Transonic Systems, Inc., Ithaca, NY, USA).

### Renal sympathetic nerve activity recording

The RSNA was recorded through the left renal nerve with bipolar platinum electrodes. After exposure and isolation, the renal nerve was placed on the recording electrodes and was covered with warm mineral oil. The RSNA signals were obtained using a high-impedance probe connected to an AC amplifier (ERS 100c; Biopac Systems, Inc., Goleta, CA, USA). The signal was amplified (20,000), passed through a band-pass filter (50–1,000 Hz) and digitized at 3 kHz using an MP150 analog-to-digital converter. The filtered nerve signal was rectified and integrated (1 s time constant) using AcqKnowledge software. At the end of each experiment, the background noise was determined as the average value of the integrated voltage after a bolus injection of hexamethonium (30 mg⋅kg^−1^ b.wt., i.v.).

### Acute increase in plasma sodium concentration

Hypertonic saline (3 M NaCl, 1.8 ml•kg^−1^ b.wt., i.v.; Sigma-Aldrich, St. Louis, MO, USA) was infused through the jugular vein cannula for 1 min.

### Blood sampling and analysis

Blood samples (200 µl each) were withdrawn from the femoral vein cannula 5 min before and 10, 30 and 60 min after HS infusion. After the sample was collected, an equal volume of sterile 0.15 M NaCl was injected to reduce changes in the extracellular fluid volume brought about by sampling. The blood hemoglobin concentration was measured immediately with a kit from Sigma (Drabkin's reagent, kit 525). The rest of the sample was centrifuged for 5 min at 6000 g. The plasma was removed and stored at −20°C. The plasma sodium concentration was measured with a flame photometer (model 943, Instrumentation Laboratory, Lexington, MA).

### Perfusion, fixation, and tissue collection

At the end of the experiments, the animals' heart were perfused with saline (0.15 M NaCl), followed by a solution of 4% paraformaldehyde (Sigma-Aldrich, St. Louis, MO, USA) in 0.1 M sodium phosphate buffer (500 ml at pH 7.4). The brain was removed and post fixed in a 4% paraformaldehyde solution for 1–2 h and was then kept in 30% sucrose solution. The serial coronal 40-µm brainstem sections were collected in four series and were stored in 0.02 M potassium phosphate-buffered saline (KPBS, Sigma; pH 7.4) at 4°C until immunohistochemical staining.

### Immunohistochemistry

Each fourth brainstem section was processed for immunohistochemical detection of tyrosine hydroxylase (TH). The sections were incubated for 15 min in 0.5% hydrogen peroxide in 0.02 M KPBS (Sigma-Aldrich, St. Louis, MO, USA), followed by a 30-min incubation in 3.5% normal horse serum (Vector Laboratories Inc., Burlingame, CA, USA) in 0.02 M KPBS. The sections were incubated overnight at 4°C with mouse monoclonal antibody (1∶2000 dilution, Immuno Star Inc., Hudson, WI, USA) with 1.5% normal horse serum and 0.2% Triton X-100 (Sigma-Aldrich, St. Louis, MO, USA), followed by a 1-h incubation with biotinylated horse anti-mouse IgG (1∶200 dilution with 0.1% Triton X-100; Vector Laboratories Inc., Burlingame, CA, USA). After these incubations, the sections were processed with the avidin-biotin procedure using Elite Vectastain reagents (Vector Laboratories Inc., Burlingame, CA, USA). Diaminobenzidine (DAB) was used to produce a brown cytoplasmic TH reaction product. The sections were set on slides, dehydrated in a series of alcohols, cleared in xylene and coverslipped.

### Cell counting and imaging

The counting of labeled neurons was performed on every fourth medulla oblongata section (40 of each 160 µm). All of the immune-labeled neuronal perikarya in the ventrolateral medulla (VLM; A1/C1) and nucleus tractus solitarius (NTS; A2/C2) were counted bilaterally to quantify the extent of the anti-DβH-saporin-induced lesion. The neurons were counted at 200× magnification with a Nikon light microscope.

### Data analysis

The changes in the RBF were expressed as a percentage of control values (means ± S.E.M). The renal vascular conductance (RVC) was calculated as the ratio of the RBF by MAP and was also expressed as a percentage of the baseline values. The changes in the RSNA were quantified by integration (1 s) after wave rectification and were expressed as percentages of the baseline after subtraction of the noise level. Trigger pulses matching the systole were used for the construction of normalized averages of the arterial pulse and the RSNA wave. For each average, a representative 100-s segment of the arterial pressure and the RSNA wave was used.

The effects of treatment with anti-DβH-saporin or saporin on the number of catecholaminergic medullary neurons are presented as means ± S.E.M. The number of cells counted for every section was compared by a one-way ANOVA. Additionally, the total cell counts for the A1, C1, A2 and C2 regions were calculated and compared between the groups by unpaired Student's t-tests. Cardiovascular, RSNA and plasma sodium and blood hemoglobin concentrations were analyzed by a two-way analysis of variance followed by the Fisher LSD test. The differences in the baseline levels (Table 1) between the groups were analyzed by unpaired Student's t-tests. A value of P<0.05 was considered to denote a significant difference.
